# Development, Validation and Primary Application of a Competency‐Based Training Course Index System for Gynaecological Specialist Nurses in China: A Mixed‐Methods Study

**DOI:** 10.1002/nop2.70476

**Published:** 2026-03-08

**Authors:** Dan Liu, Xiaoyan Peng, Tian Xiong, Pingyuan Bu

**Affiliations:** ^1^ Department of Nursing The Third Xiangya Hospital of Central South University Changsha China; ^2^ Second Ward of Interventional Vascular Endocrinology, Xiangxi Autonomous Prefecture People's Hospital Jishou China; ^3^ Department of Gynaecology The Third Xiangya Hospital of Central South University Changsha China

**Keywords:** delphi study, empirical research, gynaecological specialist nurse, post competency, training system

## Abstract

**Aim:**

The aim of this study was to establish an indexing system for training courses based on post‐competency for gynaecological specialist nurses.

**Design:**

A mixed‐methods study.

**Methods:**

We employed literature reviews and semi‐structured interviews to develop a preliminary index system. Subsequently, a two‐round Delphi consultation survey was conducted to get insights from 15 experts regarding the index for evaluating the post‐competency of gynaecological specialist nurses and to obtain qualitative feedback on their assessments. Following the finalisation of the competency index system, a 3‐month training programme was implemented with 75 specialist nurses to evaluate its practical application. These nurses completed the training and returned to their clinical duties after successfully passing the competency evaluation.

**Results:**

The comprehensive training course index system for post‐competency gynaecological specialist nurses comprises four primary indices: gynaecological‐specialised theoretical knowledge, gynaecological‐specialised practical skills, communication and coordination management skills and clinical thinking and research management skills. This system further includes 12 secondary indexes and 58 tertiary indexes. The response rates for the two expert consultation rounds were 100%. The expert authority coefficients were 0.943 and 0.942 in the initial and subsequent rounds of consultation, respectively. During the second round of consultation, the initial and subsequent indices of Kendall's coefficient of concordance were 0.210 and 0.257, respectively (*p* < 0.05). Seventy‐five specialist nurses in gynaecology completed 4 weeks of theoretical education and 8 weeks of practical training. The mean score of gynaecological specialist nurses on the theoretical examination increased from 65.4 before training to 91.2 after training (*p* < 0.001); the mean score on the self‐assessment of post‐competency rose from 39.5 prior to training to 86.8 subsequent to training (*p* < 0.001); the comparison of pre‐training and post‐training results was statistically significant.

**Conclusions:**

The index system for the training course of gynaecological specialist nurses was designed in a logical and thorough manner, and the findings from its empirical study provided a foundation for the formation of a scientific and systematic gynaecology specialist nursing team.

## 1 Introduction

Specialist nurses in gynaecology significantly enhance the quality of nursing care, ensure patient safety and foster the development of the nursing team. Currently, no research has been undertaken to develop an indexing system for training courses on post‐competency for gynaecological specialist nurses.

In this study, the term ‘training course index system’ refers to a hierarchical and structured framework of indicators. It is designed to systematically outline and standardise the essential competencies and curricular components required for a comprehensive training programme for gynaecological specialist nurses. This system serves as a blueprint for curriculum development and evaluation, ensuring that all critical aspects of post competency are covered.

## 2 Background

Women hold an increasingly significant role in both the home and society, and prioritising women's health is essential for ensuring familial and social harmony, as well as a crucial prerequisite for safeguarding the health of future generations. Gynaecological illnesses adversely impact women's health, imposing a considerable cost on their quality of life, their families and the healthcare system (Norton et al. 2020). Gynaecological disorders include but are not limited to reproductive tract infections, menstrual disorders, endocrine‐related conditions, benign and malignant tumours and infertility (Gao et al. 2025). In 2018, the prevalence of gynaecological disorders among women aged 15 and older in China was 24.94%, representing about 20% of the global total of gynaecological disease patients, indicating a tendency of under‐ageing (Ni et al. 2024). The increase in the female population, the resurgence of gynaecological illnesses and the enhancement of living standards necessitate the proper addressing of the varied needs of female patients to promote reproductive health. Nurses are essential in addressing patients' health requirements and delivering superior nursing services (Witzeman et al. 2024). Furthermore, the distinct physiological and psychological traits of women result in a greater load of psychological stress compared to patients with other ailments, significantly affecting disease prognosis and quality of life (Cook et al. 2019). This imposes more expectations on the proficiency of gynaecological nursing personnel.

Specialist nurses are registered nurses with targeted professional experience, comprehensive theoretical and practical training in their specialty and relevant qualification certificates from healthcare management agencies. They are equipped to apply professional techniques, deliver specialised nursing care to patients and offer guidance to other nursing personnel. Gynaecological specialist nurses are a subset of specialist nurses, possessing advanced theoretical knowledge and professional skills in gynaecological nursing. Specialist nurse training is essential for improving high‐quality nursing care and is a prerequisite for the evolution of nursing personnel into nursing specialists (Baxter and Edvardsson 2018). A research found that gynaecological specialist nurses could significantly reduce patients' hospital stays, enhance health outcomes and improve the overall medical experience (Dawes et al. 2007). Nonetheless, the number of gynaecological specialist nurses in China remains insufficient, and the current training system exhibits several limitations, including a lack of standardised curricula, inconsistent practical training requirements and an absence of a unified competency evaluation framework, all contributing to variations in training quality across regions. The Outline of China's Nursing Career Development Plan (2021–2025) emphasises the enhancement of specialist nurse training and the establishment of a specialised nursing workforce, with clearly defined access criteria, training prerequisites and responsibilities, to elevate the quality of specialist nursing services and address the health needs of the population (National Health Commission of the People's Republic of China 2022). Currently, gynaecological specialist nurses in China are in a nascent stage and lack a cohesive and standardised training curriculum. Consequently, China ought to actively enhance and standardise the training of gynaecological specialist nurses to address current challenges and fulfil the health requirements of gynaecological patients.

Nursing post competency, defined as the collective knowledge, skills, abilities and attributes that empower clinical nursing personnel to achieve excellence in their practice and deliver exceptional performance, is influenced by factors such as job responsibilities, work environment, incentives and constraints (Wangensteen et al. 2015). Researchers across several nations have examined the training of specialised nurses grounded in post‐competency theory, yielding consistently positive outcomes. No studies about the training course for gynaecological specialist nurses based on post‐competency in China have been identified.

In recent years, some provinces in the Chinese mainland have established training centres for gynaecological specialist nurses and launched certification programmes. However, the education, practical ability and certification of these professionals have not been standardised nationwide and there is a lack of a mature competency training course system. Although the mature models for the development of gynaecological specialist nurses in European countries and Hong Kong are of reference value, the cultures and healthcare systems vary from region to region. Therefore, it is of vital importance to construct a competency training course index system for gynaecological specialist nurses that is applicable to the medical system and cultural background of the Chinese mainland. This system will provide an objective and practical reference framework for the training of gynaecological specialist nurses in China, to enhance their job capabilities, promote the resolution of the increasingly diverse and multi‐faceted health needs of Chinese women and ultimately facilitate the systematic development of gynaecological nursing practices in China.

## 3 Materials and Methods

### 3.1 Construction of the Competency‐Based Training Course Index System for Gynaecological Specialist Nurses

#### 3.1.1 Organisation of the Research Team

The research team consisted of seven persons, including one PhD supervisor and one master's supervisor, both of whom were chief nurse practitioners. Their principal duties encompassed formulating the assessment index framework, coordinating team meetings, setting criteria for relevant experts and communicating with expert members. We assigned three gynaecological nurses, each possessing over 10 years of clinical experience, to distribute and collect questionnaires, as well as to organise and analyse the relevant data.

#### 3.1.2 Construction of the Expert Consultation Questionnaire

We performed a comprehensive literature review across widely used databases including Pubmed, Embase, Web of Science, Google Scholar, China Knowledge, Wanfang and Wipu, employing ‘gynaecology’, ‘gynaecological diseases’, ‘specialised nurses’, and ‘training’ as search keywords in both Chinese and English. The ‘Nurses' Training Syllabus in Specialized Nursing’ (National Health Commission of the People's Republic of China 2007) augmented the review. Two researchers independently reviewed the literature for eligibility and conducted data extraction. Firstly, based on literature research, we established a training index system for gynaecological specialist nurses and constructed a semi‐structured interview framework. Subsequently, we interviewed 27 nurses with more than 3 years of professional experience in gynaecology. Transcript of the interview: What outcomes do you expect from the study? What suggestions do you have about the theoretical curriculum and clinical instruction to enhance and advance specialised post‐competency? What do you consider essential for the development and enhancement of your capabilities? Do you have any suggestions concerning training and evaluation methodologies? In accordance with the idea of voluntary confidentiality, each interview lasted between 30 and 40 min. Within 24 h of the interview, we transcribed the recordings verbatim and subsequently integrated them into the qualitative analytic programme, NVivo 11.0, for data management and additional analysis. We utilised the Colaizzi seven‐step analytical procedure to scrutinise the interview data and discern themes (Ming 2019). After completing the semi‐structured interviews, we conducted cross‐validation and content supplementation on the initially constructed training index system for gynaecological specialist nurses through systematic comparison and integration of interview topics and literature evidence. The study team created a thorough repository of entries for the gynaecological nurse specialist training system following intensive discussions and the incorporation of actual realities to assess and enhance the indicators. The initial draft of the gynaecological nurse specialist training indicator system consisted of four primary indicators, 12 subsidiary indicators and 49 tertiary indicators.

#### 3.1.3 Delphi Consultation

Correspondence experts adhere to the principles of authority and representativeness. To ensure a comprehensive perspective on gynaecological specialist nurse training, the panel was composed of both nursing experts and senior gynaecological physicians. Nursing experts were drawn from the fields of nursing management, gynaecological clinical care, specialised gynaecological nursing, nursing education and nursing research. Medical experts were included given their crucial role as clinical partners in defining competency requirements and their direct involvement in nurse training and evaluation. The criteria for the inclusion of correspondence experts were: (1) holding of a master's degree or higher; (2) employment at an associate senior level or above; (3) a minimum of 15 years of experience in gynaecology; and (4) informed consent and voluntary participation. The experts participating in the Delphi survey were identified and recruited through a strategy combining purposive sampling and snowball sampling. We officially invited 15 experts to participate in the research. All 15 experts accepted the invitation and completed two rounds of surveys, indicating that the experts regarded the research topic as highly relevant and important. We distributed correspondence surveys through both online and offline methods, including on‐site delivery, email and electronic questionnaires. Experts were provided with a questionnaire containing the Competency‐Based Training Course Index System for Gynaecological Specialist Nurses. Using a Likert 5‐point scale, they were asked to rate the importance of each training indicator, while an open comments section was included to collect specific suggestions for revisions. We instructed the invited consultants to independently complete the surveys and endeavour to submit their responses within 2 weeks, so ensuring the dependability of the questionnaires and maximising the response rate. Following the initial correspondence, the study team methodically organised and analysed the collected material and expert opinions. The criteria for indicator screening required an importance score of no < 3.5, a coefficient of variation under 0.25 and a full score ratio of at least 20%. Subsequent to group discussions and expert opinions, we modified, eliminated or incorporated the indications. Following the insights from the initial expert consultation and the statistical analysis results, we formulated the questionnaire for the subsequent round of expert consultation, disseminated and structured it similarly, and concluded the consultation after the experts' perspectives aligned.

### 3.2 Application of the Competency‐Based Training Course Index System for Gynaecological Specialist Nurses

We distributed recruitment posters online to recruit nurses from across the country with more than 2 years of specialised gynaecological nursing experience. Participants were divided into three groups based on their main clinical environments: general gynaecological nurses in general hospitals, nurses in maternal and child health care hospitals and gynaecological oncology specialist nurses. These three groups of nurses respectively received a training intervention, and the changes in their working abilities were evaluated by comparing the job competency scores before and after the training. The training aims encompass registered nurses with more than 2 years of experience in gynaecological specialty nursing who necessitate a full month of theoretical standardised training and 2 months of clinical practice training. The theoretical training utilises a centralised and standardised instructional methodology, with 71% of the teaching personnel holding senior positions, 100% of the medical faculty possessing doctoral degrees or higher, and 100% of the nursing faculty having master's degrees or higher, all derived from experts associated with provincial and ministerial level hospitals. Methods of theoretical training and evaluation: (1) The programme consists of 4 weeks of specialised theoretical training, totalling 36 h each week and 4 h of apprenticeship, resulting in 160 h of theoretical instruction. (2) The programme includes essential theoretical principles of gynaecology, modern medical technology, specialised illness management, quality assurance, accelerated rehabilitation and other advanced clinical nursing theories. Furthermore, it encompasses nursing education, research methodology, health promotion, communication skills and collaborative dynamics. Individuals who successfully passed the evaluation after the theoretical training received a clinical training handbook and began the clinical practice phase. The section in charge of general training supervises six practice bases accredited by the specialty. The nursing department oversees the cohesive leadership and development of these facilities, ensuring that the educational programmes comply with established training standards. The instructional programmes are amalgamated with the theoretical elements of the clinical learning plan development. Clinical education will employ a one‐on‐one follow‐up approach, requiring teaching qualifications for nurses of competent status or above. We implement instruction via a synthesis of collective observation of standardised nursing practices, group dialogues and clinical training. During the internship at each hospital, trainees will engage in nursing assessments and discussions of complex situations, and each trainee will complete and implement one critical care case for patients. Upon completion of the internship, trainees are required to undertake a minimum of two operational assessments. After completing the necessary credits of the training programme, we will assess the learners on their theoretical and practical competencies. Those who successfully pass the assessment will be awarded a credential as a gynaecological nurse specialist by the Provincial Health Planning Commission.

### 3.3 Statistical Analysis

We utilised SPSS 25.0 programme to analyse the data. We conducted a descriptive analysis of the experts' general information utilising frequency and percentage metrics. The effective recovery rate of the correspondence questionnaire reflected the experts' favourable assessment, while the authority coefficient (Cr), derived from judgement (Ca) and familiarity (Cs), signified the experts' authority. The full marks ratio, coefficient of variation (CV) and mean value of each indicator reflected the concentration of expert viewpoints, whilst Kendall's *W* demonstrated the degree of coordination. We employed Kendall's *W* coefficient of concordance to demonstrate the alignment of expert viewpoints. To demonstrate the concentration of expert viewpoints, we utilised the mean of the important ratings for each indicator, the coefficient of variation (CV) and the overall score rate. A *p*‐value of < 0.05 was deemed statistically significant. We employed the *t*‐test and ANOVA to assess the measurement data, and the *χ* ^2^ test to evaluate the count data.

## 4 Results

### 4.1 Construction of the Competency‐Based Training Course Index System for Gynaecological Specialist Nurses

#### 4.1.1 Basic Information of the Experts

Fifteen experts participated in this consultation, comprising eight clinical nursing experts, two nursing management authorities, three medical professionals and two nursing education experts, all involved in gynaecological medical care. The nursing management experts also hold positions on provincial‐level gynaecological nursing committees.

#### 4.1.2 Expert's Topic Interest and Expert Authority Coefficient

Fifteen questionnaires were disseminated over the two rounds of expert consultation, and all 15 were retrieved, resulting in a recovery rate of 100%. This suggests that the experts exhibited heightened engagement, concern and cooperation in this phase of the research. The Cs in the initial inquiry round was 0.908, Ca was 0.977 and Cr was 0.943; in the subsequent inquiry round, Cs was 0.938, Ca was 0.946 and Cr was 0.942. The experts' degree of authority in both rounds exceeded 0.7, signifying a high level of authority among the experts in both inquiries.

#### 4.1.3 Experts' Opinion Coordination Degree

The mean importance ratings for Level 1 indicators in Round 1 of the Expert Consultation varied from 4.38 to 4.92, with coefficients of variation between 0.06 and 0.20, and full marks from 53.85% to 92.31%. The mean ratings for Level 2 indicators ranged from 4.23 to 4.92, with coefficients of variation from 0.06 to 0.22, and full marks from 38.46% to 92.31%. For Level 3 indicators, the mean ratings ranged from 4.08 to 5.00, with coefficients of variation from 0 to 0.29, and full marks from 30.77% to 100.00%. The mean importance ratings for Level 3 indicators are between 4.08 and 5.00, with coefficients of variation from 0 to 0.29, and perfect scores ranging from 30.77% to 100.00%. The indicators in the initial round of correspondence that exhibited a mean importance score exceeding 3.5, a full score rate surpassing 20%, and a coefficient of variation > 0.25 were ‘A‐1‐2 Location of blood vessels, lymphatics, and nerves’, and ‘A‐1‐3 Location and morphology of the pelvis, pelvic floor, and adjacent organs, anatomical structure and function’. The mean importance scores for primary indicators in the second round of correspondence experts ranged from 4.31 to 5.00, with coefficients of variation between 0.00 and 0.15, and perfect score percentages from 38.46% to 100.00%. For secondary indicators, mean scores ranged from 4.15 to 5.00, coefficients of variation varied from 0.00 to 0.14, and perfect score percentages spanned from 23.08% to 100.00%. Regarding Level 3 indicators, mean importance ratings ranged from 4.15 to 5.00, coefficients of variation were between 0.00 and 0.17, and perfect score percentages ranged from 23.08% to 100.00%. The average importance ratings for all indicators in the second round of correspondence exceeded 3.5, with scores above 20%, while Kendall's *W* in the first round of expert consultation was 0.210, with a significance test *p* < 0.05; Kendall's *W* in the second round of expert consultation was 0.257, with a significance test *p* < 0.05.

#### 4.1.4 Results of the Delphi Consultation Survey

The primary and secondary indicators remain unchanged, while 11 indicators have been added to the tertiary indicators, four indicators have been updated and two indicators have been removed based on expert comments and group discussions, as detailed below. (1) Under the knowledge of the pathophysiology of the female reproductive system, include ‘the ability to analyze and identify common gynecological symptoms, imaging results, and signs’; under the knowledge of gynaecological specialties, incorporate ‘knowledge of perimenopausal mental health’, ‘psychological counseling and rehabilitation of gynecological oncology patients’, ‘prevention and screening of gynecological malignant tumors’, ‘clinical techniques of assisted reproduction’, and ‘lymphoedema following treatment of gynecological oncology’. The topics ‘Prevention and treatment of lymphedema following oncology treatment’ and ‘Radiotherapy care for gynecological tumors and the prevention and treatment of complications’ will be included. Under specialised emergency evaluation ability, ‘Timely evaluation of abrupt changes in the condition of critically ill patients’ will be added. Under specialised nursing operation, ‘Familiarity with gynecological tumors’ will be incorporated. Under Specialised Nursing Practice, ‘Psychological counseling and rehabilitation’ will be included. Incorporate ‘familiarity with the surgical techniques for various gynecological conditions’ and enhance communication skills to include ‘multidisciplinary communication and coordination abilities’ as well as ‘nursing humanistic care’. (2) Modification of items: experts assert that the depiction of anatomical knowledge is imprecise, ambiguous and verbose; it is advised to revise ‘location, morphology and anatomical structure of the internal and external genitalia’ to ‘anatomy of the internal and external genitalia’, and ‘blood vessels, lymphatic and lymphatic organs, and the anatomy of the internal and external genitalia’. Consequently, ‘location, morphology and anatomical structure of internal and external genitalia’ should be amended to ‘anatomy of internal and external genitalia’, ‘location of blood vessels, lymphatic vessels and nerves’ should be revised to ‘anatomy of blood vessels, lymphatic vessels and nerves associated with the genital organs’, and ‘location, morphology, anatomical structure and function of the pelvis, the pelvic floor and the adjacent organs’ should be modified to ‘location, morphology, anatomical structure and function of the pelvic floor and adjacent organs’. Experts recommended incorporating helped reproduction into the understanding of infertility, thereby revising ‘knowledge of infertility’ to ‘knowledge of infertility and assisted reproduction’. Consequently, ‘knowledge about infertility’ was amended to ‘knowledge about infertility and assisted reproduction’. (3) Deletion of items: The expert determined that the item ‘Emergency prevention and control management of gynecological wards in case of epidemic outbreaks’ was restricted to epidemic outbreaks and redundant with ‘Emergency prevention and control management of gynecological wards in case of public health emergencies’, thus rendering the suggestion for deletion unreasonable; the expert's opinion was accepted. The experts believe that the inclusion of ‘mastering the assessment, observation, and care of symptoms of gynecological diseases’ falls outside the realm of specialised nursing operational techniques and duplicates the prior entry of ‘care and process of common acute and critical gynecological diseases’. Consequently, it is recommended for deletion, a suggestion the panel accepted after deliberation. Subsequent to deliberation, the panel endorsed this viewpoint. The gynaecological nurse training index system has four basic indicators, 12 subsidiary indicators and 58 tertiary indicators, as illustrated in Table 1.

**TABLE 1:** Consultation result of the training course system of gynaecology specialist nurses.

Competences	Mean ± SD	CV	CLV (%)
A. Theoretical knowledge of gynaecological specialties	5.00 ± 0.00	0.00	100.00
A.1. Knowledge of female reproductive system anatomy	4.77 ± 0.44	0.09	76.92
A.1.1. Anatomical structure of internal and external genitalia	4.62 ± 0.51	0.11	61.54
A.1.2. Anatomy of blood vessels, lymph and nerves related to reproductive organs	4.77 ± 0.44	0.09	76.92
A.1.3. Anatomical structure of pelvis	4.77 ± 0.44	0.09	76.92
A.2. Knowledge of the pathophysiology of the female reproductive system	4.46 ± 0.66	0.15	53.85
A.2.1. Physiological characteristics of women at different stages of their lives	4.92 ± 0.28	0.06	92.31
A.2.2. Clinical manifestations of menstruation and menstrual periods	4.77 ± 0.44	0.09	76.92
A.2.3. Ovarian function and cyclic changes	4.54 ± 0.66	0.15	61.54
A.2.4. Cyclic changes in the endometrium and other parts of the genitals	4.54 ± 0.52	0.11	53.85
A.2.5. Influence of the function of other endocrine glands on the menstrual cycle	4.85 ± 0.38	0.08	84.62
A.2.6. Ability to analyse and discriminate common gynaecologic symptoms, imaging findings and signs	4.46 ± 0.66	0.15	53.85
A.3. Knowledge of common diseases in gynaecologic specialties	5.00 ± 0.00	0.00	100.00
A.3.1. Knowledge of common inflammatory diseases of the reproductive system	4.69 ± 0.48	0.10	69.23
A.3.2. Knowledge of abnormalities of female genital development	4.69 ± 0.48	0.10	69.23
A.3.3. Knowledge related to early pregnancy complications	4.92 ± 0.28	0.06	92.31
A.3.4. Knowledge of pelvic floor dysfunction and genital injury diseases	4.85 ± 0.38	0.08	84.62
A.3.5. Knowledge of gynaecologic oncologic diseases	4.92 ± 0.28	0.06	92.31
A.3.6. Knowledge of gestational trophoblastic diseases	4.54 ± 0.52	0.11	53.85
A.3.7. Knowledge of reproductive endocrine diseases	4.77 ± 0.44	0.09	76.92
A.3.8. Knowledge of infertility and assisted reproduction	4.85 ± 0.38	0.08	84.62
A.4. Knowledge of gynaecological specialties	4.85 ± 0.38	0.08	84.62
A.4.1. Knowledge and care of high‐intensity ultrasound focusing	4.85 ± 0.38	0.08	84.62
A.4.2. Perioperative nursing routines for gynaecologic laparoscopic patients	4.92 ± 0.28	0.06	92.31
A.4.3. Perioperative nursing routines for gynaecologic pelvic floor patients	4.92 ± 0.28	0.06	92.31
A.4.4. Perioperative nursing routines for gynaecologic hysteroscopy patients	4.92 ± 0.28	0.06	92.31
A.4.5. Female sexual hygiene and sexual health education	4.38 ± 0.51	0.12	38.46
A.4.6. Nursing management of chemotherapy patients and mastering the treatment of chemotherapy drug extravasation	4.54 ± 0.52	0.11	53.85
A.4.7. Knowledge and prevention of VTE	4.54 ± 0.66	0.15	61.54
A.4.8. Family planning technical guidance and quality service	4.62 ± 0.51	0.11	61.54
A.4.9. Knowledge of basic diseases closely related to gynaecology	4.69 ± 0.48	0.10	69.23
A.4.10. Perimenopausal mental health related knowledge	4.77 ± 0.44	0.09	76.92
A.4.11. Psychological counselling and rehabilitation of gynaecological oncology patients	4.46 ± 0.52	0.12	46.15
A.4.12. Prevention and screening of gynaecological malignant tumours	4.46 ± 0.52	0.12	46.15
A.4.13. Clinical techniques of assisted reproduction	4.85 ± 0.38	0.08	84.62
A.4.14. Prevention and treatment of lymphedema after gynaecologic tumour treatment	5.00 ± 0.00	0.00	100.00
A.4.15. Gynaecological tumour radiotherapy nursing and complication prevention and treatment	5.00 ± 0.00	0.00	100.00
B. gynaecology specialty practice skills	5.00 ± 0.00	0.00	100.00
B.1. Specialty emergency assessment skills	4.92 ± 0.28	0.06	92.31
B.1.1. Nursing care and process of common emergency and critical illnesses in gynaecology	5.00 ± 0.00	0.00	100.00
B.1.2. Timely assessment in the face of sudden changes in the condition of critically ill patients	4.62 ± 0.65	0.14	69.23
B.2. Specialised nursing operation skills	4.92 ± 0.28	0.06	92.31
B.2.1. First‐aid skills and gynaecological first‐aid drill	4.54 ± 0.66	0.15	61.54
B.2.2. Specialised operation techniques	5.00 ± 0.00	0.00	100.00
B.2.3. Gynaecological surgery and nursing care	4.54 ± 0.52	0.11	53.85
B.2.4. Knowledge of abdominal heat perfusion therapy and emergency treatment during the operation process	4.46 ± 0.52	0.12	46.15
B.2.5. Evaluation, observation and care of various tubes in gynaecology specialties	4.54 ± 0.52	0.11	53.85
B.2.6. Surgical approaches to various diseases in gynaecology	4.92 ± 0.28	0.06	92.31
C. Communication and coordination management skills	4.31 ± 0.63	0.15	38.46
C.1. Professionalism	4.85 ± 0.38	0.08	84.62
C.1.1. Nursing consultation skills and difficult case discussion skills	4.77 ± 0.44	0.09	76.92
C.1.2. Interpersonal communication methods and skills	5.00 ± 0.00	0.00	100.00
C.1.3. Multidisciplinary communication and coordination ability	4.15 ± 0.69	0.17	30.77
C.1.4. Nursing humanistic care	4.92 ± 0.28	0.06	92.31
C.2. General competence	4.15 ± 0.55	0.13	23.08
C.2.1. Nursing management of gynaecological day surgery patients	4.69 ± 0.48	0.10	69.23
C.2.2. Risk management of gynaecological patients	4.69 ± 0.48	0.10	69.23
C.2.3. Emergency prevention and control management of gynaecology wards in public health emergencies	4.77 ± 0.44	0.09	76.92
C.2.4. Establishment and Continuous Improvement of Sensitive Quality Indicators in Gynaecology Specialties	4.77 ± 0.44	0.09	76.92
D. Clinical thinking and research management skills	4.54 ± 0.52	0.11	53.85
D.1. Nursing diagnosis and clinical thinking ability	4.92 ± 0.28	0.06	92.31
D.1.1. Case nursing	4.15 ± 0.69	0.17	30.77
D.1.2. Timely nursing diagnosis based on patient's actual condition	4.23 ± 0.44	0.10	23.08
D.1.3. Critical thinking	4.15 ± 0.69	0.17	30.77
D.2. Learning competencies	4.77 ± 0.44	0.09	76.92
D.2.1. Problem‐based simulation learning	4.69 ± 0.48	0.10	69.23
D.2.2. Scenario‐based simulation of specialty skill operations	4.54 ± 0.52	0.11	53.85
D.3. Research competency	4.23 ± 0.60	0.14	30.77
D.3.1. Nursing literature search and management of literature	4.38 ± 0.65	0.15	46.15
D.3.2. Research paper writing	4.23 ± 0.60	0.14	30.77
D.4. Teaching ability	4.31 ± 0.48	0.11	30.77
D.4.1. clinical teaching methods and techniques	4.62 ± 0.51	0.11	61.54
D.4.2. Conducting evidence‐based teaching	4.31 ± 0.48	0.11	30.77
D.4.3. Competence in health science education and promotion	4.85 ± 0.38	0.08	84.62

### 4.2 Application of the Competency‐Based Training Course Index System for Gynaecological Specialist Nurses

No statistically significant difference was seen among the specialist nurses regarding age, years of experience, educational background and professional title (*p* > 0.05), as illustrated in Table 2.

**TABLE 2:** General information of gynaecology specialist nurses in three groups (*N* = 75).

Items	Group I (*N* = 27)	Group II (*N* = 26)	Group III (*N* = 22)	*F*	*p*
Age	29.88 ± 4.12	31.88 ± 4.12	30.18 ± 5.82	5.50	0.006
Working years	7.08 ± 4.92	8.45 ± 6.55	7.59 ± 6.41	1.42	0.248
Education background (bachelor degree and above)	100%	100%	90%		
Professional title (Nurse or nurse practitioner and above)	100%	100%	100%	*χ* ^2^ = 0.184	0.912

#### 4.2.1 Comparison of Differences in Theoretical Scores and Post Competence for Gynaecological Specialist Nurses

The theoretical scores of the specialist nurses in each group post‐training were markedly higher than those pre‐training, with a statistically significant difference (*p* < 0.001). After training, the self‐assessment scores of specialised nurses in each group regarding post competency were significantly elevated compared to pre‐training values, with a statistically significant difference (*p* < 0.001), as outlined in Table 3.

**TABLE 3:** Comparison of differences in theoretical scores and post competence among three groups of gynaecology specialist nurses (*N* = 75).

Group	Theoretical scores^a^			Post competence^a^		
	Group I (*N* = 27)	Group II (*N* = 26)	Group III (*N* = 22)	Group I (*N* = 27)	Group II (*N* = 26)	Group III (*N* = 22)
Before the training	65.4 ± 9.6	62.9 ± 12.9	66.2 ± 7.8	39.5 ± 2.9	41.9 ± 5.67	44.2 ± 5.4
After the training	90.6 ± 4.5	89.7 ± 9.7	91.2 ± 3	77.6 ± 3.2	81.1 ± 6.42	86.8 ± 4.56
*t*	24.70	16.93	28.06	51.68	46.67	56.54
*p*	< 0.001	< 0.001	< 0.001	< 0.001	< 0.001	< 0.001

*Note:* The self‐assessment of post competence has a total of 27 items, including four dimensions: professional competence, knowledge competence, general competence and professionalism, with a score range of 0–108 points.

^a^ Theoretical score out of 100 points.

## 5 Discussion

### 5.1 Reliability and Scientific Validity of Results

Gynaecology encompasses a diverse array of conditions, including benign and malignant neoplasms, inflammatory disorders of the reproductive system and infertility, among others. Annually, gynaecological patients require numerous consultations, frequent hospitalisations and present with intricate conditions, resulting in a significant demand for the proficiency of gynaecological nurses in clinical practice, education, scientific research and overall quality assurance (Jie et al. 2021; Yan et al. 2022). This study established the initial draft of the training index system for gynaecological specialist nurses using a literature review and semi‐structured interviews, with the final system being refined after two rounds of expert correspondence. The experts involved in the consultation hailed from gynaecological medicine, nursing and nursing education, with 84.7% holding the status of associate senior or higher, indicating strong representation. During the two rounds of consultation, the response rate for the questionnaires was 100.00%. In the first round, 38.46% of the experts provided suggestions, indicating a high level of engagement and concern for the study among the participating experts. This indicates that the experts exhibited greater engagement in the study and shown a higher level of interest and collaboration. The authority coefficients from the two rounds of expert consultation in this study are 0.943 and 0.942 (both > 0.7), signifying a high level of expert authority. The mean value, coefficient of variation and percentage of full marks of the experts' importance evaluations for each indicator level reflect the concentration of experts' viewpoints. A decreased coefficient of variation, coupled with a larger mean value and a higher percentage of full marks, signifies more importance of the indicator and a heightened concentration of experts' viewpoints. In the initial round of expert consultation, the mean importance ratings for each indicator varied from 4.08 to 5.00 points, with a coefficient of variation between 0.00 and 0.29, and a percentage of full marks ranging from 30.77% to 100.00%. In the subsequent round, the mean ratings ranged from 4.15 to 5.00 points, the coefficient of variation was between 0.00 and 0.17, and the percentage of full marks varied from 23.08% to 100.00%. During the second round of expert consultation, the mean importance score for each indicator varies between 4.15 and 5.00 points, the coefficient of variation spans from 0.00 to 0.17, and the total score ranges from 23.08% to 100.00%, demonstrating a high degree of consensus among the experts. Kendall's *W* quantifies the extent of agreement among experts' viewpoints. Kendall's *W* values are generally close to 1, suggesting a higher consistency among experts' opinions and improved coordination. The Kendall's *W* for the two rounds of expert correspondence is 0.210 and 0.257, respectively, with statistically significant differences (*p* < 0.05), indicating enhanced coordination of expert opinions. Consequently, the index system for gynaecological specialist nurse training developed in this study demonstrates robust scientific validity.

### 5.2 Analysis and Significance of the Training Course System Contents

The development of the gynaecological specialist nurse training index system encompasses theoretical knowledge, practical abilities and teaching and research competencies in gynaecology. Among these, sufficient theoretical knowledge is fundamental to ensuring the core competency of specialised nurses (Li et al. 2018). Post‐competency communication and coordination management skills are developed and enhanced through education. This study delineates the theoretical knowledge specialty training content across four dimensions: anatomy of the female reproductive system, pathophysiology, prevalent gynaecological diseases and gynaecological specialty‐related knowledge, thereby facilitating systematic and comprehensive training for gynaecological specialty nurses. Specialised practical skills involve the application of theoretical knowledge, serving as a crucial benchmark for evaluating professional competency (Huang et al. 2018). This study's content on specialised training in practical skills encompasses specialised emergency assessment capabilities and specialised nursing operational techniques. Training in specialised emergency assessment equips nurses to respond swiftly and decisively in emergencies, thereby enhancing their emergency response proficiency. Additionally, training in specialised nursing operational techniques further augments the emergency response capabilities of specialised nurses (Guo et al. 2021). Training in nursing operation technology can raise the proficiency and precision of specialised nursing procedures, facilitate the delivery of accurate and prompt nursing interventions for patients in clinical settings, so elevating the quality of nursing services and increasing patient satisfaction. The establishment of the gynaecology specialist nurse training index system transforms the prior model of knowledge and skill acquisition, broadening the scope of specialised training to encompass professionalism and general competencies. By considering the unique attributes of the trainees, we develop tailored training plans that address their deficiencies and emphasise key training areas, thereby enhancing training efficiency and achieving superior outcomes. This study posits that the ability to manage communication and coordination encapsulates the essence of specialised humanistic nursing. Effective communication and coordination are vital components of the nurse–patient relationship; proficient nurse–patient communication skills can significantly mitigate patients' negative psychological states, foster a harmonious nurse–patient rapport and consequently enhance the efficacy of clinical care and patient satisfaction (Wu et al. 2017). The components of professional literacy and general competency encompass four elements: clinical reasoning, humanistic literacy, coordination skills and management proficiency, which enhance specialised nurses' capacity to adeptly use professional knowledge and evaluate and resolve issues (Wang et al. 2021). To enhance the clinical applicability of the gynaecology specialist training index system, a continuous improvement process will be established, creating a research chain of ‘construction‐validation‐application‐improvement’, thereby facilitating the clinical transformation and application of research findings (Xue et al. 2020).

### 5.3 Limitations

The Delphi study aimed at developing the index system for gynaecological specialty nurse training courses has certain drawbacks. The expert consultation was vulnerable to subjective influences and may have lacked substantial theoretical and logical reasoning. Secondly, the experts originated from 13 cities across eight provinces and municipalities (Beijing, Shanghai, Hubei, Guangdong, Shandong, Jilin, Shaanxi and Hunan), potentially lacking representativeness. Consequently, in the subsequent study, we will investigate the impact of implementing training courses across various geographical contexts.

## 6 Conclusion

This study developed a training course system for gynaecology specialist nurses, grounded in their clinical competencies. It comprises four core courses: basic knowledge in gynaecology specialisation, practical skills in gynaecology, communication and coordination management skills and clinical thinking and scientific research skills. The programme encompasses the development of clinical skills related to gynaecology nursing, teaching, scientific research, vocational literacy and general competency, among other post‐competences (Cheng et al. 2019). The gynaecological specialist nurse training course was developed systematically and fully, with its empirical results providing a platform for the formation of a scientific and organised team of gynaecological specialist nurses.

## Author Contributions

**Dan Liu:** conceptualisation, methodology, investigation, writing original draft. **Xiaoyan Peng:** investigation, data collection, methodology. **Tian Xiong:** methodology, data collection, formal analysis. **Pingyuan Bu:** supervision, writing – review and editing, project administration. All authors contributed to the study conception and design.

## Funding

The authors have nothing to report.

## Ethics Statement

The research process followed the principles of voluntariness, secrecy and ethical criteria, including adherence to the Declaration of Helsinki. The initiative was approved by the Ethics Review Committee (No. 24980). The clinical trial number is not applicable. The study followed ethical guidelines, including obtaining informed consent, maintaining confidentialityand demonstrating respect for the autonomy and privacy of participants.

## Conflicts of Interest

The authors declare no conflicts of interest.

## Data Availability

De‐identified data that support the findings of this study are available on request from the corresponding author. The data are not publicly available due to privacy and ethical restrictions.
